# First-principles calculated decomposition pathways for LiBH_4_ nanoclusters

**DOI:** 10.1038/srep26056

**Published:** 2016-05-18

**Authors:** Zhi-Quan Huang, Wei-Chih Chen, Feng-Chuan Chuang, Eric H. Majzoub, Vidvuds Ozoliņš

**Affiliations:** 1 Department of Physics, National Sun Yat-Sen University, Kaohsiung 804, Taiwan; 2 Center for Nanoscience and Department of Physics and Astronomy,University of Missouri-St. Louis, St. Louis, Missouri 63121, United States; 3 Department of Materials Science and Engineering, University of California Los Angeles, Los Angeles, California 90095-1595, USA

## Abstract

We analyze thermodynamic stability and decomposition pathways of LiBH_4_ nanoclusters using grand-canonical free-energy minimization based on total energies and vibrational frequencies obtained from density-functional theory (DFT) calculations. We consider (LiBH_4_)_*n*_ nanoclusters with *n* = 2 to 12 as reactants, while the possible products include (Li)_*n*_, (B)_*n*_, (LiB)_*n*_, (LiH)_*n*_, and Li_2_B_*n*_H_*n*_; off-stoichiometric Li_*n*_B_*n*_H_*m*_ (*m *≤ 4*n*) clusters were considered for *n* = 2, 3, and 6. Cluster ground-state configurations have been predicted using prototype electrostatic ground-state (PEGS) and genetic algorithm (GA) based structural optimizations. Free-energy calculations show hydrogen release pathways markedly differ from those in bulk LiBH_4_. While experiments have found that the bulk material decomposes into LiH and B, with Li_2_B_12_H_12_ as a kinetically inhibited intermediate phase, (LiBH_4_)_*n*_ nanoclusters with *n* ≤ 12 are predicted to decompose into mixed Li_*n*_B_*n*_ clusters via a series of intermediate clusters of Li_*n*_B_*n*_H_*m*_ (*m* ≤ 4*n*). The calculated pressure-composition isotherms and temperature-pressure isobars exhibit sloping plateaus due to finite size effects on reaction thermodynamics. Generally, decomposition temperatures of free-standing clusters are found to increase with decreasing cluster size due to thermodynamic destabilization of reaction products.

Hydrogen is a promising next-generation energy carrier due to its high energy density, high energy conversion efficiency, and absence of harmful emissions. However, on-board hydrogen storage is still a serious challenge for developing economically viable hydrogen-powered passenger vehicles^1^. To achieve widespread commercialization, hydrogen storage systems should simultaneously possess several characteristics such as safety, high gravimetric and volumetric densities, fast reversible hydrogen release and uptake under moderate pressures and temperatures matched to the operating conditions of proton exchange membrane (PEM) fuel cells.

Complex metal hydrides emerged as feasible high-density hydrogen storage materials after Bogdanović and Schwickardi demonstrated reversible (de)hydrogenation reactions in transition metal doped sodium alanate (NaAlH_4_)^2^. Spurred by this discovery, many other complex hydrides have been explored as candidate hydrogen storage materials, including alanates, amides, and borohydrides. Lithium borohydride, LiBH_4_, has received particular attention owing to its high gravimetric (18.5 wt.% H_2_) and volumetric (121 g H_2_/L) densities. However, due to its high thermodynamic stability and slow kinetics, hydrogen release from bulk LiBH_4_ requires very high temperatures that are incompatible with proton-exchange membrane (PEM) fuel cells^3^,^4^,^5^. In addition, the tendency to release diborane during dehydrogenation leads to irreversibility and causes poisoning of the fuel cell^6^,^7^,^8^,^9^,^10^.

Destabilization has been extensively explored as a means of improving the thermodynamics of hydrogen storage reactions^11^,^12^,^13^,^14^. The main idea of this approach is to add a second reactant, which upon decomposition forms a stable low-energy product phase and lowers the overall reaction enthalpy. The canonical example of a destabilized reaction is LiBH_4_ + MgH_2_ → LiH + MgB_2_ + (5/2)H_2_, where the addition of MgH_2_ leads to the formation of MgB_2_ as a low-energy product phase. This significantly lowers the reaction enthalpy relative to the decomposition reaction of the pure compound, LiBH4 → LiH + B + (3/2)H_2_. Unfortunately, destabilization is not very effective in lowering the *kinetic* barriers to hydrogen release, leading to only modest improvement in the hydrogen release temperatures.

Another approach to improving the thermodynamics and kinetics of hydrogen release consists of using LiBH_4_ nanoparticles incorporated in support materials with nanoscale pores, such as nanoporous carbon or metal-organic frameworks (MOFs)^9^,^10^,^15^,^16^,^17^,^18^,^19^,^20^,^21^,^22^,^23^,^24^. Besides improving the kinetics and lowering the temperature of hydrogen release, this strategy has also been shown to suppress the formation of diborane^19^,^25^,^26^. First-principles density-functional theory (DFT) based studies^27^,^28^ have investigated the thermodynamic properties of nano-LiBH_4_, suggesting that bonding with the nanoporous carbon support plays a key role in improving the thermodynamic properties of nano-LiBH_4_. However, due to the large configuration space, these studies did not perform comprehensive examination of the most stable cluster structures and compositions as functions of cluster size. Hence, a complete picture of the size-dependent thermodynamics properties of LiBH_4_ nanoparticles and their decomposition products is not yet available.

Decomposition pathways and pressure-composition isotherms are determined using free energy minimization in the grand canonical ensemble^29^. In addition to clusters with the chemical composition of bulk LiBH_4_ and its predicted bulk decomposition products Li_2_B_12_H_12_, LiH and B^30^, we considered (Li)_*n*_, (LiB)_*n*_, and Li_2_B_*n*_H_*n*_ clusters as possible products. To assess the role of other cluster compositions, further detailed studies were performed for the decomposition reactions of (LiBH_4_)_2_, (LiBH_4_)_3_ and (LiBH_4_)_6_ including Li_2_B_2_H_*n*_ (*n* = 1 to 7), Li_3_B_3_H_*n*_ (*n* = 1 to 11) and Li_6_B_6_H_*n*_ (*n* = 1 to 23) clusters as possible intermediate products. Our analysis shows that the reaction end products are changed from (LiH)_*n*_ + B_*n*_ + (3/2)H_2_ to (LiB)_*n*_ + 2*n*H_2_ when the size of reactants is reduced to the nanoscale. The calculated reaction enthalpies decrease from 238 (*n* = 2) to 133 kJ/mol H_2_ (*n* = 12) with increasing cluster size, remaining significantly above the bulk decomposition enthalpy of LiBH_4_. The hydrogen release temperatures are predicted to behave in a non-trivial way, with multiple intermediates appearing in the simulated dehydrogenation processes. The finite size effects and presence of intermediates manifest in multiple plateaus in the calculated release curves as functions of temperature, while the calculated pressure-composition isotherms and temperature-composition isobars exhibit sloping plateaus. Our study provides an in-depth understanding of the thermodynamics of hydrogen release from nanoscale particles and shows the importance of engineering appropriate support materials that can bind the reaction products and lower the reaction enthalpies to achieve reversible hydrogen storage in a practically viable range of temperatures and pressures.

## Results

### Structure of reactants and products

**Figure 1:**
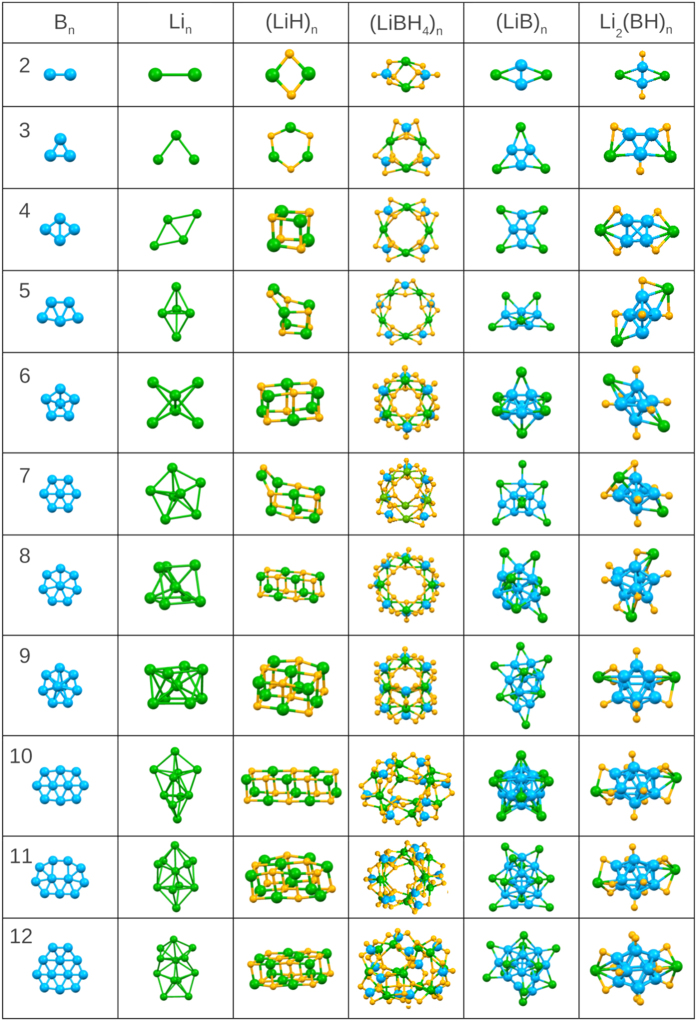
First-principles relaxed cluster geometries found in this work. B, Li, and H atoms are the blue, green and yellow spheres, respectively.

We start by briefly describing our findings for the atomic geometries of the reactants and products, which are summarized in Fig. 1. The reactants, LiBH_4_ nanoclusters, are composed of the cation, Li^+^, and the anionic group, 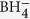. We find that the lowest energy conformations of small (LiBH_4_)_*n*_ clusters are chainlike, forming closed rings. The tetrahedral 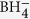 anion is a near perfect tetrahedron in all cases with some elongation in the B-H bond length from 1.22 to 1.24 Å. Note that the length of the B-H bond in bulk LiBH_4_ is 1.22 Å. The diameter of LiBH_4_ clusters increases gradually from 6.0 to 10.4 Å upon increasing the cluster size from *n* = 2 to 12. (LiH)_*n*_ clusters form rocksalt-derived structures for sizes *n* ≥ 4.

For boron clusters, our GA method reproduced the lowest energy structures found in earlier studies^31^,^32^,^33^. Small boron clusters are found to form 2-dimensional planar structures except for the B_9_ cluster which has two boron atoms on two sides of the circular substructure with seven boron atoms forming a ring. Changes in the cluster geometries with increasing number of boron atoms indicate that boron nanoclusters prefer to have a coordination number of six.

For pure lithium clusters, the GA again finds the same structures as those in previous studies^34^,^35^. Unlike boron clusters, lithium clusters agglomerate in three-dimensional motifs when the the cluster sizes exceed four atoms.

In the mixed lithium-boron clusters (LiB)_*n*_, the boron atoms generally tend to gather in the center of the cluster with lithium atoms surrounding them on the periphery. In addition, for *n* ≤ 5 the arrangement of boron atoms is the same as in the pure boron clusters. When *n* > 5, boron atoms in the center are distorted due to interactions with the surrounding lithium atoms. We also found that the B-B distances in the center of the (LiB)_*n*_ clusters are gradually increased from 1.53 Å for *n* = 2 to 1.85 Å for *n* = 12, while the Li-B bond lengths stay approximately constant. It appears that interactions with the outer Li ions and charge transfer from Li to B combine to push the B atoms outward.

### Cluster energies

**Figure 2:**
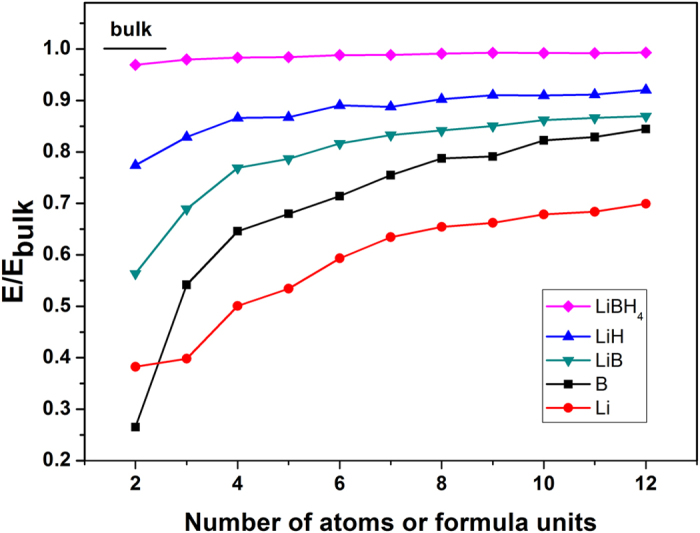
Calculated DFT total energies (without zero-point energy corrections) of (LiBH_4_)_*n*_, (LiH)_*n*_, (LiB)_*n*_, B_*n*_ and Li_*n*_ clusters, normalized to the corresponding bulk values.

To better understand the thermodynamic stability, reaction pathways and reaction enthalpies, we first discuss the cluster-size dependent energetics of reactants and reaction products. Figure 2 shows the relative energies of clusters normalized by their bulk energies without the zero-point energy (ZPE) corrections. This quantity is defined as the ratio of the energy of the cluster per formula unit versus the energy of the bulk phase. Bulk lithium is calculated in the 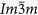 body-centered cubic lattice, bulk boron is in the *α*-B phase, and bulk LiH is in the rocksalt structure. Bulk LiBH_4_ has several near-degenerate ground-state structures^36^, and the *Pnma* structure is used in our calculations^37^. As for bulk LiB, we used a *P*6_3_/*mmc* structure reported in a previous experimental study^38^. The calculated reaction enthalpy for the decomposition of bulk LiBH_4_ at *T* = 0 K is Δ*H* = 60 kJ/(mol H_2_) with zero-point energy (ZPE) corrections, while excluding ZPE we obtain 82 kJ/(mol H_2_). The predicted decomposition temperature is T_dec_ = 340 °C at *p* = 1 bar hydrogen pressure, which is about 100 °C below experimental results. We find that the stabilities of the reaction products are dramatically reduced with decreasing cluster size, with LiH and LiB clusters being more stable than pure Li and B clusters. However, the behavior of the reactant LiBH_4_ clusters is very different: the energy per formula unit of (LiBH_4_)_*n*_ is nearly flat from *n* = 2 to *n* = 12, which signals that their thermodynamic stability is reduced only slightly upon decreasing cluster size. This suggests that most of the binding energy is stored in the polar covalent B-H bonds, while the electrostatic interactions between lithium and 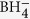 complexes are relatively weak. Examination of the size-dependent stabilities of each cluster type shows that clusters with even-numbered formula units are generally more stable than the odd ones. For pure clusters, Li_*n*_ and B_*n*_, we find that Li_3_, Li_5_, Li_9_, Li_11_, B_7_, B_9_, and B_11_ possess certain geometric symmetries, but they are unstable with respect to decomposition into even-numbered *n* + 1 and *n* − 1 clusters due to unpaired electron filling in the highest occupied molecular orbital. As for the LiH and LiB clusters, even though the highest orbitals in (LiH)_5_, (LiH)_7_, (LiH)_11_, and (LiB)_9_ are fully occupied, these clusters are unstable because of broken geometric symmetry. Moreover, (LiH)_10_ and (LiB)_5_ are also unstable because they are at the structural transition point between distinct bonding topologies.

### Decomposition pathways of LiBH_4_ clusters

Previous computational work^30^ has shown that, thermodynamically, decomposition of bulk LiBH_4_ should occur via the formation of an intermediate closoborane Li_2_B_12_H_12_ compound. However, the formation of closoboranes is kinetically inhibited in experiments and decomposition proceeds directly to a mixture of LiH and B. We begin our discussion of the decomposition pathways of LiBH_4_ nanoclusters by considering simplified decomposition reactions with closoborane Li_2_B_*n*_H_*n*_, elemental (Li)_*n*_ and (B)_*n*_, and binary (LiH)_*n*_ and (LiB)_*n*_ clusters as the end products; the full treatment using the ensemble grand canonical formalism is given below. First, we consider the following reaction pathways:


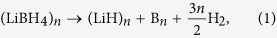



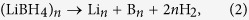



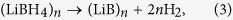



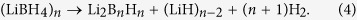


Equations 1 and 2 are the nanocluster analogues of the bulk decomposition reactions occurring at *T* = 400 and 900 °C, respectively^3^. Reaction 4 is the nanocluster analogue of the bulk decomposition reaction into Li_2_B_12_H_12_, accounting for the formation of various closoborane (B_*n*_H_*n*_)^2−^ species with *n* ≤ 12, while the product of Eq. 1 can be obtained by further decomposition of a mixture of Li_2_B_*n*_H_*n*_ and (LiH)_*n*−2_. Finally, Eq. 3, is similar to the favored decomposition reaction predicted for nano-NaAlH_4_^29^.

**Figure 3:**
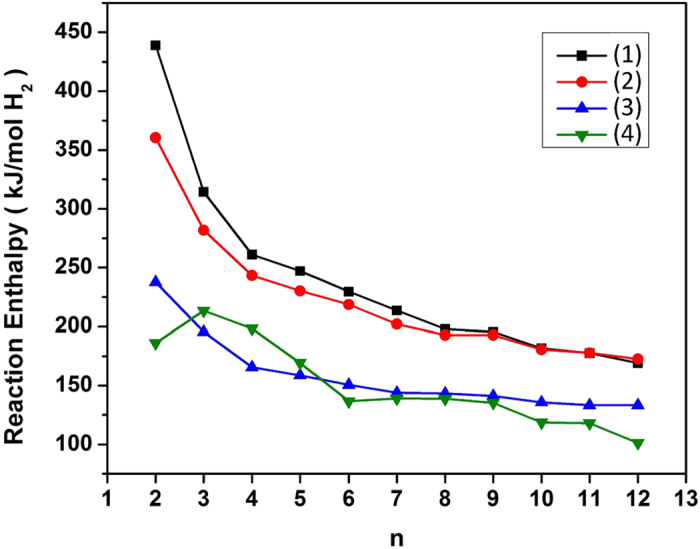
Calculated DFT reaction enthalpies(without ZPE correction) of the four decomposition pathways of nano-LiBH_4_ given by Eqs 1–4Eqs 1–4Eqs 1–4Eqs 1–4, shown as functions of the cluster size *n*.

The calculated DFT reaction enthalpies of Eqs 1, 2, 3, 4 without vibrational free energy contributions are shown in Fig. 3. In all cases, the calculated DFT reaction enthalpies are larger than the decomposition enthalpy of bulk LiBH_4_ [82 kJ/(mol H_2_)]. This does not mean that LiBH_4_ nanoclusters are generally more stable with respect to decomposition than the bulk compound because these data are based on a limited number of possible reactions and reaction products; we will show below that the Gibbs free energy minimization approach and inclusion of product clusters with intermediate hydrogen content can lower the reaction enthalpies significantly. The trend towards higher reaction enthalpies for the hypothesized reactions in Eqs 1, 2, 3, 4 can be easily explained from the size-dependence of the cluster binding energies in Fig. 2: since the (LiBH_4_)_*n*_ clusters show weak size dependence, while all the product clusters show much stronger finite size energy penalty, the reaction enthalpies are significantly higher than the bulk limit. It is interesting to note that of all four reaction pathways, the bulk-like Eq. 1 is the least favorable one in nano-LiBH_4_, which shows that the reaction path can change dramatically upon reducing the particle size to the nanometer regime; similar effects have been predicted for sodium alanate^29^,^39^. Both pathways in Eqs 2 and 3 liberate all the hydrogen, and our results show that Eq. 3 is more favorable than Eq. 2 because the binary (LiB)_*n*_ clusters are more stable than the mixture of (Li)_*n*_ and (B)_*n*_ clusters. We note that the formation of Li_2_B_*n*_H_*n*_ clusters via Eq. 4 is the preferred path for *n* = 6 to 12 when closoborane species form as the end product, while the pathway in Eq. 3 is preferred for n = 3 to 5. In general, we find that the calculated reaction enthalpies decrease with increasing cluster size *n*, and are significantly higher than the calculated DFT enthalpies for decomposition of bulk-LiBH_4_ into Li_2_B_12_H_12_^30^. Due to the high stability of 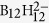 complex anions, the reaction enthalpy of Eq. 4 drops significantly from *n* = 11 to *n* = 12, and one may expect that these species will persist as the dominant end product for *n* > 12.

**Figure 4:**
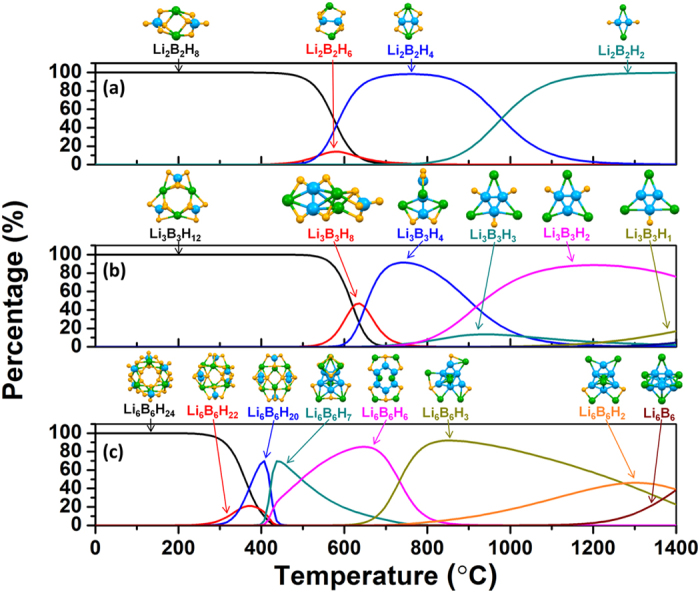
Calculated decomposition pathways of (**a**) (LiBH_4_)_2_, (**b**) (LiBH_4_)_3_, and (**c**) (LiBH_4_)_6_ nanoclusters obtained from Gibbs free energy minimization, Eqs S1–S3. Lithium, boron and hydrogen atoms are shown as green, blue, and yellow spheres, respectively.

To investigate the possibility that intermediate products other than those in Eqs 1, 2, 3, 4 may form during decomposition, we have carried out calculations for clusters with the general formula Li_*n*_B_*n*_H_*m*_, where 0 < *m* < 4*n* and *n* = 2, 3, and 6. For this series, we adopted the random search scheme rather than the genetic algorithm. The random search scheme is implemented as follows. New initial structures were randomly generated by removing one or more H atoms from nano-LiBH_4_ and by gradually adding H atoms to LiB nanoclusters, breaking symmetries by slightly distorting all atoms away from the original positions. These randomly generated structures were then relaxed using DFT forces to a local minimum configuration, and the energies of the most favorable structures at each composition were used in the Gibbs ensemble formalism given by Eqs S1–S3 (See Supplementary Information). The zero point energy, temperature-dependent vibrational free energy, and the free energy of the diatomic H_2_ gas are included in these calculations. Our results for the cluster type fractions *p*_*f*_ are summarized in Fig. 4 assuming a hydrogen pressure of *p* = 0.01 bar, which is representative of experimental measurements.

Figure 4(a) shows the calculated dehydrogenation pathway of (LiBH_4_)_2_ and the structures of the predicted intermediate products as functions of temperature. (LiBH_4_)_2_ nanoclusters start to release hydrogen at approximately 450 °C by first forming Li_2_B_2_H_6_. The structure of this cluster resembles diborane, with a significant difference that the donation of two electrons from Li atoms reconfigures the three-center two-electron bonds of diborane into a direct B-B bond. At 500 °C, Li_2_B_2_H_4_ clusters form and exist as the dominant specie from 580 to 980 °C. Finally, Li_2_B_2_H_2_ clusters appear at 800 °C and dominate until full dehydrogenation into (LiB)_2_ as the end product above 1400 °C (not shown). We note that the intermediate products in the dehydrogenation pathway of (LiBH_4_)_2_ all contain an even number of hydrogen atoms, and each intermediate reaction step releases one H_2_ molecule per cluster.

Figure 4(b) shows the decomposition pathway of (LiBH_4_)_3_. It is seen that hydrogen release starts at 530 °C where two H_2_ molecules per cluster are released to form Li_3_B_3_H_8_, which exists in a narrow temperature interval. The latter cluster is essentially a superposition of Li_2_B_2_H_4_ and (LiBH_4_)_1_ clusters, which explains its marginal stability. In the next step, two more H_2_ molecules are released to form Li_3_B_3_H_4_, bypassing Li_3_B_3_H_6_ entirely. At temperatures of 920 °C and higher, a Li_3_B_3_H_2_ cluster is formed by releasing another H_2_ molecule, before full dehydrogenation to the end product Li_3_B_3_. Small fraction of Li_3_B_3_H_3_ and Li_3_B_3_H_1_ clusters with odd-numbered hydrogen atoms is predicted to appear intermittently.

Finally, Fig. 4(c) shows the dehydrogenation pathway of (LiBH_4_)_6_. Even-numbered Li_6_B_6_H_22_ and Li_6_B_6_H_20_ clusters stage a brief appearance at 250 and 300 °C, respectively. The structures of these clusters can be described as derived from the parent borohydride cluster by releasing a hydrogen molecule and forming a 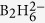 complex anion. It is tempting to hypothesize that 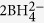 → 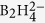 also represents a *kinetically* viable hydrogen release pathway, but this has to be verified by separate studies of the activation energies. In any case, the negative charge carried by the 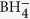 anions should present a significant barrier to bringing these complexes together for the reaction. Most of the hydrogen is released around 420 °C with the appearance of Li_6_B_6_H_7_ and Li_6_B_6_H_6_ clusters. The latter cluster exhibits a high degree of symmetry and appears to be stable over a wide range of temperatures up to approximately 700 °C. In the final reaction steps, Li_6_B_6_H_3_, Li_6_B_6_H_2_ and Li_6_B_6_ show up one by one as the majority cluster phases. Again, it is seen that most of the stable clusters in the decomposition sequence contain an even number of hydrogen atoms, with the brief appearance of Li_6_B_6_H_7_ as the only exception.

**Figure 5:**
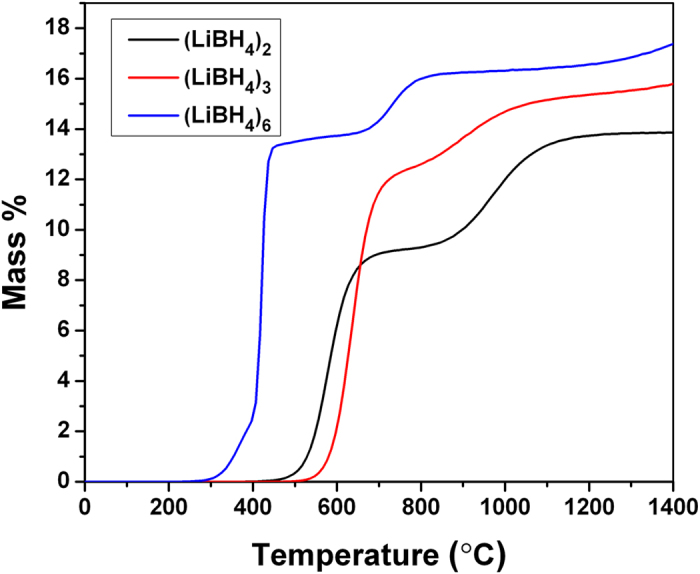
Mass fraction of released hydrogen as a function of temperature at constant hydrogen pressure *p* = 0.01 bar.

Based on the data in Fig. 4, we have calculated the cumulative mass fraction of released H_2_ as a function of temperature at constant pressure (i.e., temperature-composition isobar). Figure 5 shows the results of these calculations. It is seen that the calculated hydrogen release curves exhibit nontrivial behavior as functions of temperature and cluster size.

For instance, the initial onset of hydrogen release occurs at a lower temperature for the *n* = 2 cluster than for the *n* = 3 cluster. This is due to the fact that the *n* = 2 cluster can release one H_2_ molecule and form Li_2_B_2_H_6_ (which consists of two Li^+^ ions and a 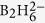 anion), while the corresponding Li_3_B_3_H_10_ compound is unfavorable because it involves a mixture of 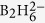 and 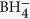 anions. Furthermore, all three clusters exhibit two sloping plateaus in their dehydrogenation curves. Focusing on the *n* = 6 cluster, most of the hydrogen comes off in a narrow temperature interval before the first plateau, corresponding to successive removal of hydrogen molecules and eventual formation of Li_2_B_6_H_6_ with an overall formula Eq. 4. In the second stage, a smaller amount of hydrogen is released by decomposing Li_2_B_6_H_6_ and forming Li_2_B_6_H_3_, etc. With increasing cluster size of nano-LiBH_4_, we expected that the amount of hydrogen released in the first-stage will increase and that the main hydrogen release step will shift to lower temperatures. According to the reaction enthalpies in Fig. 3, the overall reaction for *n* ≤ 12 is expected to proceed according to Eq. 4, even though it seems likely that the full pathway will consist of multiple intermediate steps, similar to those discussed above. For *n* > 12, we hypothesize that the formation of clusters with Li-compensated 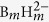 complex anions (6 ≤ *m* ≤ 12) will be favorable, but the existence of intermediate steps is also likely, in a manner similar to the *n* = 6 case. Unfortunately, reliable structure prediction for such large systems is beyond the capabilities of current computational methods.

Due to the increased enthalpy difference between the reactants and products, to achieve dehydrogenation at the same pressure as bulk LiBH_4_, (LiBH_4_)_6_ nanoclusters need temperatures that are approximately 200 °C higher. Since experimental measurements on nanoconfined borohydrides^18^,^19^,^20^ often find lowering of the desorption temperature, this indicates that the supporting substrate plays an important role by binding the supported nanoclusters and lowering the enthalpy of the reaction products relative to (LiBH_4_)_*n*_.

**Figure 6:**
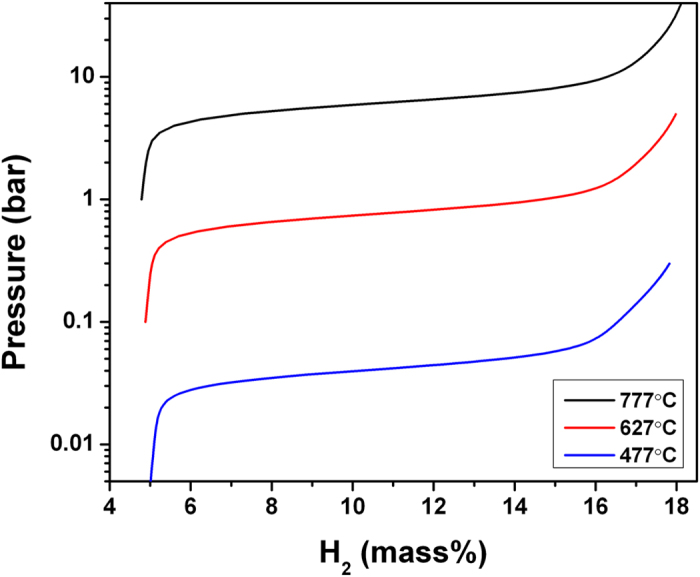
Calculated pressure-composition isotherms for (LiBH_4_)_6_ clusters.

While dehydrogenation is usually carried out by increasing temperature at a constant pressure, as shown in Figs 4 and 5, hydrogen absorption measurements are commonly done by increasing pressure at constant temperature. Hence, pressure-composition isotherms (PCT), showing the equilibrium pressure of the H_2_ gas coexisting with a material with a given the hydrogen content, are of considerable interest. For bulk materials, these curves show flat regions corresponding to equilibria between the hydrogen gas and two solid phases on the opposite sides of the miscibility gap. In nanomaterials, clusters with different hydrogen content play the role of the coexisting solid phases. However, in contrast to bulk systems where the plateaus are perfectly flat in the thermodynamic limit with no kinetic limitations, in finite size clusters the plateaus acquire slope. This is illustrated in Fig. 6, which shows the calculated DFT pressure-composition isotherms for (LiBH_4_)_6_ clusters at three different temperatures. The left-hand side is the dehydrogenated phase, Li_6_B_6_H_6_, corresponding 4.6 wt.% H_2_ on the horizontal axis. The fully hydrogenated phase, (LiBH_4_)_6_ is on the right-hand side, at 18.5 wt.% H_2_. Going right-to-left, the sloping plateaus describe the series of increasingly hydrogen-poor clusters in the decomposition sequence of (LiBH_4_)_6_, with the slope arising from the intermediate steps and from the fact that the cluster probabilities *p*_*f*_ in Eq. S2 vary continuously with pressure and temperature. We note that sloping plateaus are seen in experimental study of pressure-composition isotherms of bulk-LiBH_4_ 5, but this is likely due to kinetic limitations, not thermodynamic effects. Additionally, the apparent width of the plateau increases slightly with increasing temperature, which we attribute to the narrowing of the thermodynamic stability windows for the intermediate cluster compositions between (LiBH_4_)_6_ and Li_2_B_6_H_6_.

## Conclusions

We present a detailed computational study of the decomposition thermodynamics of small LiBH_4_ nanoclusters using DFT-based ground state structure prediction algorithms and total DFT free energies, including vibrational contributions. With a few exceptions at very small sizes, nanoclusters favor the same decomposition sequence into closoborane Li_2_B_*n*_H_*n*_ species as bulk LiBH_4_. The calculated reaction enthalpies are higher than those for bulk materials because the total formation energies of the reaction product clusters approach their bulk values significantly slower than (LiBH_4_)_*n*_. Hence, reduction of cluster size is not expected to improve the thermodynamic properties of LiBH_4_ for use in reversible hydrogen storage. Since experimental studies have demonstrated significant reduction of hydrogen release temperatures in supported nanoclusters, this confirms the importance of cluster-substrate binding energies for the thermodynamics of hydrogen release reactions. It also suggests that technologically favorable thermodynamics of LiBH_4_ nanocluster decomposition could be engineered by varying the microscopic structure and composition of the porous substrate to lower the enthalpies of the reactants and achieve hydrogen release at temperatures compatible with the operating conditions of PEM fuel cells. Furthermore, we find that in all cases the decomposition pathways of (LiBH_4_)_2_, (LiBH_4_)_3_, and (LiBH_4_)_6_ consist of multiple intermediate stages, most of them separated by release of an integer number of H_2_ molecules. While in bulk systems each reaction occurs along a sharp temperature-pressure curve given by the van’t Hoff relation, in nanoclusters the transitions between the cluster phases of different compositions are smooth and characterized by broad coexistence regions on the temperature and pressure; within these regions, clusters of different compositions coexist and their equilibrium fractions vary continuously. As a result, the calculated pressure-composition isotherms exhibit sloping plateaus and smooth variation near the regions of stable bulk compositions.

### Computational Methods

The structural and thermodynamic properties of small (LiBH_4_)_*n*_ (*n* ≤ 12) clusters are studied using first-principles DFT^40^,^41^ calculations in conjunction with state-of-the-art genetic algorithm (GA)^42^,^43^,^44^ and prototype electrostatic ground state search (PEGS)^45^ for structure predictions. The detailed descriptions of our computational methods can be found in the Supplementary Information. Decomposition pathways and pressure-composition isotherms are determined using free energy minimization in the grand canonical ensemble (see Supplementary Information).

## Additional Information

**How to cite this article**: Huang, Z.-Q. *et al.* First-principles calculated decomposition pathways for LiBH_4_ nanoclusters. *Sci. Rep.* **6**, 26056; doi: 10.1038/srep26056 (2016).

## Supplementary Material

Supplementary Information
