# Supplementary material for: First-principles calculated decomposition pathways for LiBH4 nanoclusters
Source: Sci Rep. 2016 May 18;6:26056. doi: 10.1038/srep26056 (PMC4870692; doi:10.1038/srep26056)
Supplement: Supplementary Information [file srep26056-s1.pdf]

Supplementary Information for: First-principles calculated decomposition pathways for $LiBH_4$ nanoclusters

Zhi-Quan Huang,[1,*] Wei-Chih Chen,[1,*] Feng-Chuan Chuang,[1,†] Eric H. Majzoub,[2] and Vidvuds Ozoliņš[3,‡]

[1]*Department of Physics, National Sun Yat-Sen University, Kaohsiung 804, Taiwan*

[2]*Center for Nanoscience and Department of Physics and Astronomy, University of Missouri-St. Louis, St. Louis, Missouri 63121, United States*

[3]*Department of Materials Science and Engineering, University of California Los Angeles, Los Angeles, California 90095-1595, USA*

[*] These two authors contributed equally.

[†] fchuang@mail.nsysu.edu.tw

[‡] vidvuds@ucla.edu

Computational Methods

Cluster Geometries

To predict decomposition paths and reaction thermodynamics, we first need to predict the ground state geometries of $LiBH_4$ nanoclusters and candidate reaction products. We considered $(LiB)_n$, $(LiH)_n$, Li_n, B_n and $Li_2B_nH_n$ as possible reaction products for all n. The method for predicting the geometries of these nanoclusters will be described below. Since small lithium and boron clusters have been investigated theoretically by several authors, we have recalculated the boron clusters B_n(n = 2 to 12) based on the the structures reported in the literature.[1–3] The structures of the ionically bonded $(LiBH_4)_n$ and $(LiH)_n$ clusters were obtained via prototype electrostatic ground state search (PEGS),[4] while the metallic clusters (Li_n and $(LiB)_n$) were obtained via genetic algorithm (GA). Since $(LiH)_n$ clusters were found to form cutouts of the rocksalt structure, possible cluster topologies were enumerated manually and relaxed using the conjugate-gradient method and forces obtained from first-principles DFT calculations. We adopted the conformations of $Li_2B_nH_n$ clusters with $n \geq 6$ from a previous study.[5]

PEGS for $(LiBH_4)_n$ clusters

The reactant clusters, $(LiBH_4)_n$ were generated using the PEGS method described in ref.[4], which has been used successfully to find several ground-state structures, high-temperature polymorphs and metastable structures in ionic materials.[6–9] In PEGS, one minimizes the electrostatic energy of a system of positively and negatively charged ion complexes, with no *a priori* symmetry constraints. Since covalent and metallic interactions are not explicitly included, PEGS *per se* is not expected to provide accurate energetics, and first-principles DFT calculations are always used to calculate the energies of the candidate ground state structures predicted by PEGS. The Hamiltonian is given by

$$H = \sum_{i<j} \left(\frac{Q_i Q_j}{r_{ij}} + \frac{\epsilon_{ij}}{r_{ij}^{12}} \right),$$

where Q_i are the ionic charges, r_{ij} are the distances between ions, and a soft-sphere repulsion term is nonzero when the inter-ionic distance is smaller than the sum of the ionic

radii. Complex ions are represented as rigid objects with fixed bond lengths and bond angles. Lithium borohydride clusters were modeled as a collection of Li^+ cations and $[LiB_4]^-$ tetrahedral anions with fixed B-H bond lengths of $R_{\text{B-H}}$=1.23 Å and vertex ionic charges $Q_{\text{H}} = +0.7$. We used ionic radii $R_{\text{Li}} = 0.68$ Å and $R_{\text{H}} = 1.24$ Å; the choice of the ionic radius for B is immaterial because it is surrounded by a tetrahedron of H ions.

In order to find the structures of $(LiBH_4)_n$ clusters, our implementation utilizes the uniform histogram Wang-Landau (WL) algorithm[10] to efficiently search the configuration space. Due to the fact that the WL method uses an estimate of the density of states to accept or reject trial moves, it can avoid being trapped in local energy minima and hence explore all possible configurations with energies below a given upper bound. A detailed implementation of PEGS using the WL algorithm is given in ref.[11], which describes application of the method to obtain ionic $(NaH)_n$, $(AlH_3)_n$, and $(NaAlH_4)_n$ nanoclusters with $n \leq 8$. Each WL run produces many topologically distinct candidate structures with low electrostatic energies; these structures are relaxed using DFT and the lowest-energy structure is selected as the ground state configuration.

Genetic algorithm for metallic clusters

Since the PEGS Hamiltonian is not expected to provide a reasonable description of the energetics of metallic clusters, a different approach was adopted for Li_n and Li_nB_n clusters, where we resorted to a genetic algorithm. Our genetic algorithm has been successfully used in global structural optimizations for C_{60},[12] medium-sized silicon clusters,[13] metal clusters,[14] and the reconstructions of crystal surfaces.[15–18] In the present study, our implementation is similar to our previous study for pure Al clusters.[14] Three types of operations including mating, mirror, and mutation, have been used to generate new structures in our GA optimization. In the mating operation, two parent structures A and B are randomly picked from the population pool and randomly rotated about their centers of mass which are set to be the origin. Then, a child structure is generated by mating the lower-half of parent A with the upper-half of parent B. If the number of atoms in the new structure does not match with that in the parent, parents A and B are shifted along the z-axis in order to achieve the correct match. In the *mirror* operation, a parent structure is picked from the population pool and rotated randomly about its center of mass which is set to be the origin.

This parent is shifted along the z-axis in order to balance the number of atoms in both halves. An offspring is created simply by duplicating the lower-half into the upper-half. The new upper-half is shifted along the z-axis to reach an appropriate distance between the two halves. Finally, in the third operation, *mutation*, a parent structure is picked from the population pool and the outmost atoms of this cluster are randomly removed and then recapped on the surface of the cluster. The mutation operation was used in our previous study[19,20] and is useful for studying doping in the cluster.

In order to maintain diversity, it is important to apply a structural comparison algorithm when updating the population pool. Our structural comparison algorithm is based on a contact matrix[21] rather than the computationally demanding method used previously in ref.[14]. Note that similar but different isomers may have the same contact matrix which will be excluded from the generation pool. Thus, we imposed a condition that if the child structure has an energy difference greater than 30 meV/atom compared to a structure with the same contact matrix, we still insert the new child structure into the populations provided that the child structure has a sufficiently low energy to be included in the pool.

The genetic algorithm optimizations in the present study were performed as follows. In generation zero, a population pool of $p = 50$ structures for a given cluster size n were generated by putting n atoms randomly in a cubic simulation box of moderate side length (see details in the next section). The structures were relaxed to their local minima using a first-principles DFT calculation with the steepest descent method and a lower cut-off energy. In each subsequent generation, one new structure was generated from three types of operations (i.e., mating, mutation, and rotation as described above) with a given probability distribution: 80% of chance for mating, 10% of chance for mutation, and 10% for rotation operations. These new structures were relaxed to a local minimum configuration by a first-principles DFT calculation. The population pool was updated if the newly relaxed structures were not identical to any isomers inside the population pool and have lower energies. Genetic algorithm optimizations were produced for up to 1000 generations.

At the end of the global minimum structure search, as many as $p = 50$ structures remain as possible candidates. All candidates are further optimized using accurate first-principles DFT calculations with a high cut-off energy and a larger simulation box (see below).

First-principles DFT calculations

First-principles DFT calculations were carried out within the generalized gradient approximation (GGA) to the exchange-correlation functional [22–24] using projector-augmented-wave pseudopotentials[25] as implemented in the *Vienna Ab-initio Simulation Package*.[26,27] Two different settings were used for different accuracies. When the VASP is coupled with our genetic algorithm, the kinetic energy cut-off is set to be 239 eV and non-spin-polarized calculation is performed. The length of the simulation box is set to the diameter of a cluster plus 8 Å with a smaller box size. The structural optimization is done with the steepest descent method and without symmetry until the forces on the atoms are less than 0.01 eV/Å.

At the end of a GA run, the kinetic energy cut-off is increased to 398.3 eV and spin-polarized calculation is performed. For clusters containing less than 8 formula units, the length of simulation cell is set to 15 Å. For $n > 9$, the supercell length is 20 Å. After the structures are determined, we use a higher kinetic energy cut-off equal to 875 eV, and the structural optimization is done with the conjugate gradient algorithm and without symmetry until the forces on the atoms are less than 0.001 eV/Å. This setting with a high accuracy was used for all the clusters in this study (shown in Fig. 1 from main text) including those obtained from PEGS and from the literature. After precise electronic structure calculations, we performed vibrational frequency calculations using the PHONOPY package.[28] We ensure the mechanical stability of all the clusters by checking the phonon frequencies for imaginary values. Temperature dependent vibrational free energies are obtained by summing contributions from all phonon modes, excluding rigid translations and rotations.

We further examined whether the candidate structures from the GA coupled with VASP with a lower accuracy setting underwent substantial relaxations upon first-principles optimizations. We found that the calculations using both low and high accurate settings retain the same energy trends and atomic structures. In order to ensure that the ground state structures for large clusters are not missed, we have chosen, for each cluster size, all 50 candidate structures in the final GA pool as the initial structures for our first-principles optimization using a higher accuracy.

Cluster phase diagrams and decomposition pathways

Theoretical prediction of hydrogen storage reactions in small clusters requires extension of the grand-canonical linear programming (GCLP) framework, which was developed for bulk materials by Akbarzadeh *et al.* in their treatment of the Li-Mg-N-H system.[29] GCLP uses the Gibbs free energy of a system in contact with a reservoir of gaseous hydrogen to determine the equilibrium phases at a given temperature and pressure. For each temperature, one scans over the compositions of non-hydrogen species (i.e., Li and B in this study) and minimizes the Gibbs free energy to determine equilibrium phase fractions of all solid phases that are consistent with the given concentration of Li and B (hydrogen content is allowed to vary). Changes in the phase fractions at two successive temperatures are used to rigorously identify all thermodynamically reversible hydrogen storage reactions.[7,30,31] In bulk systems, the unknown phase fractions are real numbers between 0 and 1, and the minimization of the Gibbs free energy can be performed using standard linear programming methods. However, linear programming cannot be used to find the equilibrium states of finite clusters due to discreteness of the composition space. Furthermore, statistical fluctuations in the number of hydrogen atoms play an important role in finite clusters and lead to the coexistence of clusters with different compositions and hydrogen content. These phenomena are taken into account in the modified ensemble formalism described below.

Consider an ensemble of finite systems, each containing one or more clusters with a total fixed atom count N_s of non-hydrogen species s (i.e., Li and B); the number of hydrogen atoms can vary since each system can exchange hydrogen with a reservoir of gaseous hydrogen with chemical potential $\mu_{\mathrm{H}}(p,T)$. In each system, the constituent atoms are allowed to assume any configuration that consists of clusters of different sizes, as long as they sum up to the required values of N_s. Mathematically, each possible configuration f is described by a set of positive integer variables y_i^f, which gives the number of clusters of type i in configuration f (typically, for thermodynamically stable configurations these numbers assume values 0 and 1). Mathematically, the constraints on the allowed configurations f are:

$$N_s = \sum_i y_i^f n_i^s \text{ and } y_i^f \geq 0, \tag{S1}$$

ensuring that the total number of all non-hydrogen atoms is fixed. Feasible solutions to Eq. S1 form a finite set, which can be enumerated using integer programming techniques

(i.e., using *Wolfram Mathematica's* built-in function `FindInstance`). The probability of finding any given configuration f is then given by

$$p_f = \frac{1}{Z(p,T)} \exp\left\{-\beta \sum_i y_i^f \left[F_i(T) - \mu_{\mathrm{H}}(p,T) n_i^{\mathrm{H}}\right]\right\}, \tag{S2}$$

where $\beta = 1/k_B T$ and $F_i(T)$ is the free energy of cluster i, including vibrational contributions (pressure dependence of cluster free energies can be neglected). The number of hydrogen atoms in the cluster i is given by n_i^{H}, and the chemical potential of gaseous hydrogen as a function of pressure and temperature is given by $\mu_{\mathrm{H}}(p,T)$. The partition function $Z(p,T)$ is calculated by summing over all configurations f that are consistent with the constraints given by Eq. S1:

$$Z(p,T) = \sum_f \exp\left\{-\beta \sum_i y_i^f \left[F_i(T) - \mu_{\mathrm{H}}(p,T) n_i^{\mathrm{H}}\right]\right\}. \tag{S3}$$

---

1. Boustani, I. Systematic ab initio investigation of bare boron clusters:mDetermination of the geometry and electronic structures of B_n (n=2–14). *Phys. Rev. B* **55**, 16426 (1997).
2. Nguyen, M. T., Matus, M. H., Ngan, V. T., Grant, D. J. & Dixon, D. A. Thermochemistry and Electronic Structure of Small Boron and Boron Oxide Clusters and Their Anions. *J. Phys. Chem. A* **113**, 4895 (2009).
3. Tai, T. B., Grant, D. J., Nguyen, M. T. & Dixon, D. A. Thermochemistry and Electronic Structure of Small Boron Clusters (B_n, n = 5–13) and Their Anions. *J. Phys Chem A* **114**, 994 (2010).
4. Majzoub, E. H. & Ozoliņš, V. Prototype electrostatic ground state approach to predicting crystal structures of ionic compounds: Application to hydrogen storage materials. *Phys. Rev. B* **77**, 104115 (2008).
5. Ohba, N. *et al.* First-principles study on the stability of intermediate compounds of $LiBH_4$. *Phys. Rev. B* **74**, 075110 (2006).
6. Ozoliņš, V., Majzoub, E. H. & Wolverton, C. First-Principles Prediction of a Ground State Crystal Structure of Magnesium Borohydride. *Phys. Rev. Lett.* **100**, 135501 (2008).
7. Ozoliņš, V., Majzoub, E. H. & Wolverton, C. First-Principles Prediction of Thermodynamically

Reversible Hydrogen Storage Reactions in the Li–Mg–Ca–B–H System. *J. Am. Chem. Soc.* **131**, 230 (2009).

8. Majzoub, E. H. & Rönnebro, E. C. Crystal Structures of Calcium Borohydride: Theory and Experiment. *J. Phys. Chem. C* **113**, 3352 (2009).
9. Seballos, L., Zhang, J. Z., Rönnebro, E., Herberg, J. L. & Majzoub, E. H. Metastability and crystal structure of the bialkali complex metal borohydride $NaK(BH_4)_2$. *J. Alloys Compd.* **476**, 446 (2009).
10. Wang, F. & Landau, D. P. Determining the density of states for classical statistical models: A random walk algorithm to produce a flat histogram. *Phys. Rev. E* **64**, 056101 (2001).
11. Majzoub, E. H., Zhou, F., & Ozoliņš, V. First-Principles Calculated Phase Diagram for Nanoclusters in the Na-Al-H System: A Single-Step Decomposition Pathway for $NaAlH_4$. *J. Phys. Chem. C* **115**, 2636 (2011).
12. Deaven, D. M. & Ho, K. M. Molecular Geometry Optimization with a Genetic Algorithm. *Phys. Rev. Lett.* **75**, 288 (1995).
13. Ho, K. M. *et al.* Structures of medium-sized silicon clusters. *Nature* **392**, 582 (1998).
14. Chuang, F. C., Wang,C. Z. & Ho, K. M. Structure of neutral aluminum clusters Al_n ($2 \leq n \leq 23$): Genetic algorithm tight-binding calculations. *Phys. Rev. B* **73**, 125431 (2006).
15. Chuang, F. C., Ciobanu, C. V., Shenoy, V. B., Wang, C. Z. & Ho, K. M. Finding the reconstructions of semiconductor surfaces via a genetic algorithm. *Surf. Sci.* **573**, L375 (2004).
16. Chuang, F. C., Ciobanu, C. V., Predescu, C., Wang, C. Z. & Ho, K. M. Structure of Si(114) determined by global optimization methods. *Surf. Sci.* **578**, 183 (2005).
17. Chuang, F. C., Ciobanu, C. V., Wang, C. Z. & Ho, K. M. Model reconstructions for the Si(337) orientation. *J. Appl. Phys.* **98**, 073507 (2005).
18. Chuang, F. C., Liu, B., Wang, C. Z., Chan, T. L. & Ho, K. M. Global structural optimization of Si magic clusters on the Si(111) 7×7 surface. *Surf. Sci. Lett.* **598**, L339 (2005).
19. Chuang, F. C., Hsu, C. C., Hsieh, Y. Y. & Albao, M. A. Atomic and Electronic Structures of $AuSi_n$ (n = 1–16) Clusters: A First-Principles Study. *Chin. J. Phys.* **48**, 79, (2010).
20. Chuang, F. C., Hsieh, Y. Y., Hsu, C. C. & Albao, M. A. Geometries and stabilities of Ag-doped Si_n (n = 1–13) clusters: A first-principles study. *J. Chem. Phys.* **127**, 144313 (2007).
21. McKay, B. D. Practical Graph Isomorphism. *Congr. Numer.* **30**, 45 (1981).
22. Hohenberg, P. & Kohn, W. Inhomogeneous Electron Gas. *Phys. Rev.* **136**, B864 (1964).

23. Kohn, W. & Sham, L. J. Self-Consistent Equations Including Exchange and Correlation Effects. *Phys. Rev.* **140**, A1133 (1965).

24. Perdew, J. P., Burke, K. & Ernzerhof, M. Generalized gradient approximation made simple. *Phys. Rev. Lett.* **77**, 3865 (1996).

25. Kresse, G. & Joubert, D. From ultrasoft pseudopotentials to the projector augmented-wave method. *Phys. Rev. B* **59**, 1758 (1999).

26. Kresse, G. & Hafner, J. *Ab initio* molecular dynamics for liquid metals. *Phys. Rev. B* **47**, 558 (1993).

27. Kresse, G. & Furthmüller J. Efficient iterative schemes for *an initio* total-energy calculations using plane-wave basis set. *Phys. Rev. B* **54**, 11169 (1996).

28. Togo, A., Oba, F. & Tanaka, I. First-principles calculations of the ferroelastic transition between rutile-type and $CaCl_2$-type SiO_2 at high pressures. *Phys. Rev. B* **78**, 134106 (2008).

29. Akbarzadeh, A. R., Ozoliņš, V. & Wolverton, C. First-Principles Determination of Multicomponent Hydride Phase Diagrams: Application to the Li–Mg–N–H System. *Adv. Mater.* **19**, 3233 (2007).

30. Alapati, S. V., Johnson, J. K. & Sholl, D. S. Large-Scale Screening of Metal Hydride Mixtures for High-Capacity Hydrogen Storage from First-Principles Calculations. *J. Phys. Chem. C* **112**, 5258 (2008).

31. Michel, K. J., Akbarzadeh, A. R. & Ozoliņš, V. First-Principles Study of the Li–Mg–N–H System: Compound Structures and Hydrogen-Storage Properties. *J. Phys. Chem. C* **113**, 14551 (2009).
